# Development and Validation of a Self‐Perception Tool for Measuring Nurse Managers’ Crisis Leadership

**DOI:** 10.1155/jonm/3754050

**Published:** 2025-12-06

**Authors:** Sun Ju Kim, Abdulqadir J. Nashwan

**Affiliations:** ^1^ Hemodialysis Department, Chungnam National University Sejong Hospital, 20 Bodeum 7-ro, Sejong-si, 30099, Republic of Korea

**Keywords:** crisis leadership, instrument, nurse managers, psychometrics

## Abstract

During crises, nurse managers’ leadership can significantly affect the quality of nursing care. Therefore, we aimed to develop a crisis leadership tool for nursing managers. This study involved nurse managers from 34 hospitals in South Korea. The crisis leadership tool was developed by reviewing the relevant literature and existing measurement tools. Five factors were identified through exploratory and confirmatory factor analyses, with confirmatory factor analysis showing goodness of fit (comparative fit index = 0.92, Tucker–Lewis index = 0.91, root mean square error of approximation = 0.07, standardized root‐mean‐square residual = 0.02). The final tool comprised 28 items across five factors: “crisis management competency” (12 items), “decision‐making” (6 items), “collaboration” (4 items), “communication” (3 items), and “building trust” (3 items), all organized on a 5‐point Likert scale. The tool’s Cronbach’s *α* was 0.96. The crisis leadership tool showed strong psychometric characteristics on four validity scales and high internal reliability. This tool can quantitatively measure the crisis leadership levels of nurse managers and may be useful in future research and practice.

## 1. Introduction

A sudden increase in the number of inpatients leads to a significant increase in the demand for nurses, highlighting the crucial role of nurse managers in efficiently managing workloads and departmental functions [1]. Nurse managers possess a high level of clinical expertise and are responsible for administrative tasks, inventory management, risk and operational management, and healthcare quality control [2]. Additionally, they oversee healthcare quality system management while adhering to professional regulations and organizational policies and procedures. Nurse managers must continually develop their expertise while fulfilling roles in education, coaching, mentoring, supervision, and counseling [3]. Choi et al. [4] found that the competence of nurse managers can significantly influence the work environment, subsequently affecting nurses’ job satisfaction and retention. This competence also positively impacts the performance of healthcare organizations or departments regarding staff management, outcome evaluation, team composition, delegation, conflict or problem resolutions, and change control [5].

Despite the crucial role of nurse managers, who are required to perform multidimensional leadership tasks [6], previous studies, have shown that newly hired nurse managers receive almost no education, support, or feedback regarding their management skills [7]. They often lack leadership skills owing to inadequate formal education and support from the hospital organizations, resulting in role stress [8]. Therefore, nursing organizations in healthcare are making continual efforts to establish and implement professional development programs for nurses to enable nurse managers to acquire the competencies crucial for effective leadership [9].

Leadership is vital during crises [10] and in acute healthcare environments. For example, nurse managers’ leadership capabilities are crucial aspects because they must organize, manage, and take responsibility for nursing activities in patient care [11]. In times of crisis, nurse managers support response measures with their skills and knowledge regarding the dynamics, pharmacology, psychology, and cultural background of the survivors and their families [12]. Additionally, resources are scarce, and conditions constantly change in a crisis, making management challenging and dangerous; however, leaders must adapt [13] and possess suitable crisis leadership skills and capabilities [14].

Crises, including the COVID‐19 pandemic, are associated with increased levels of anxiety, depression, post‐traumatic stress disorder, psychological distress, and stress among nurses. In addition to the healthcare crisis and increased mortality, these crises negatively affect society and the economy [15, 16]. Previous studies indicate that inadequate management by nurse managers during a crisis can adversely affect the quality of patient care and facilitate the spread of infection [17]. Thus, effective crisis leadership of nurse managers is essential, particularly in a healthcare environment, for ensuring immediate patient safety, reducing mortality, and addressing the challenges posed by the crisis.

Crisis leadership tools for nurse managers provide consistent standards and a structural foundation that can help them develop personal competencies to prepare for their role as a nursing manager in future crises as well as clarify the content and level of crisis leadership [18].

Several crisis leadership tools, including the Crisis Leader Efficacy in Assessing and Deciding (C‐LEAD) scale, have been developed and explored [19]. However, this scale was developed for civilian entrepreneurs, people involved in public/government organizations, and personnel in nonprofit/charitable organizations. Moreover, other tools such as the Implementation Leadership Scale (ILS) [20] and Multifactor Leadership Questionnaire [21], which measure general leadership, are commonly used in acute care settings or mental health hospitals. In our literature search for the psychometric properties of crisis leadership using the PubMed, ScienceDirect, Web of Science, and CINAHL search engines, we encountered challenges in identifying tools specifically designed to measure crisis leadership within a healthcare context.

Therefore, in this study, we aimed to analyze the crisis leadership properties of nurse managers and develop a tool to clarify the content and level of crisis leadership, providing basic information on the standardization of crisis leadership education.

## 2. Materials and Methods

### 2.1. Study Design

In this methodological study, we developed a tool to measure the level of crisis leadership of nurse managers and examined its validity and reliability.

### 2.2. Participants

We used convenience sampling to select 34 general and advanced general hospitals in South Korea that approved participation in the study between August 1 and October 29, 2023. We then selected the head nurses, team leaders, directors of nursing, and department managers from these hospitals who agreed to participate in the study. The sample size was based on the recommendation of 10 times the number of parameters required for the main statistical approach involving confirmatory factor analysis [22].

### 2.3. Research Procedure

To develop and validate items in the crisis leadership measurement tool, our study was divided into two stages—tool development and validation.

### 2.4. Tool Development

All linguistic processes, including interviews and item development, were conducted in Korean.

#### 2.4.1. Theoretical Framework

Leadership refers to the ability of a leader to influence personal or group activity to achieve the goals of an organization [23]. In the nursing field, leadership is the ability and skill to lead one’s team, solve problems appropriately, and act as a shield or support system for their team [24]. In a concept analysis study of nursing leadership, the five properties of “personal growth, cooperation, outstanding nursing, creative problem‐solving, and influence” were identified [25].

Crises in healthcare settings extend beyond infectious disease outbreaks and encompass a wide range of unexpected, disruptive events, including natural disasters (e.g., earthquakes and floods), social conflicts, wars, and systemic failures. These situations are characterized by sudden increments in patient volume, disruptions to resource availability, impaired communication with central command structures, and difficulties in staff mobilization due to infrastructure breakdowns or safety threats. In such scenarios, nurses and nurse managers are required to make rapid decisions with limited information, manage scarce resources, and lead interprofessional teams under significant psychological and physical stress. Therefore, crisis leadership in nursing must address the multifaceted challenges associated with both clinical care and organizational coordination during these extreme conditions [26, 27].

**Figure 1:**
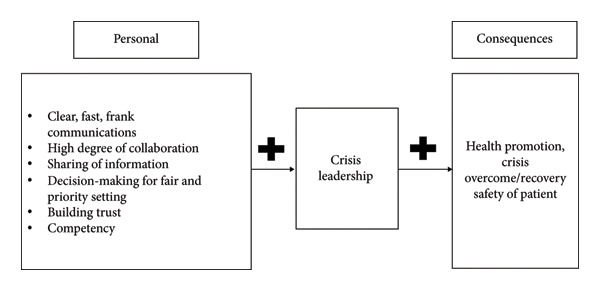
Theoretical framework.

The Middle East respiratory syndrome (MERS) pandemic was an infectious disease outbreak that led to crisis situations in many hospitals, and nurse managers played an important role during this crisis [26]. Therefore, we first developed the following theoretical framework, based on a qualitative study of nurse managers during the MERS pandemic [26] and a concept analysis of crisis leadership [27]. In our study, we defined crisis leadership as actions taken by nursing leaders to overcome a crisis by combining clear, rapid, and honest communication; decision‐making ability; and competencies based on fair prioritization to build trust, enhance cooperation and information sharing, and improve or restore the health of patients and work conditions of nurses. Crisis leadership was divided into six factors: “communication,” “decision‐making,” “collaboration,” “information sharing,” “trust,” and “competencies.” Clear, rapid, and honest communication in a crisis, cooperation, information sharing, decision‐making, trust building, and competencies are positively related to crisis leadership. A high level of crisis leadership can improve health outcomes, help the staff overcome and recover from the crisis, and enhance patient safety (Figure 1).

#### 2.4.2. Development of Preliminary Items

We developed items for each factor in the theoretical framework and prepared 38 preliminary items with help from a team leader in an infection control center at a general hospital. For the first round of content validation, the preliminary items were reviewed by a six‐person expert panel consisting of one director of nursing, three nursing team leaders, and two head nurses in May 2023. The panel rated whether each item was valid for the concept being measured, using the following scoring system: 4 points for “very valid,” 3 points for “valid,” 2 points for “not valid,” and 1 point for “not at all valid.” We also collected opinions about items that were difficult to understand or needed revisions. Based on a combination of the panel review and the opinions of two professors at a college of nursing, we edited the items, including changes to ambiguous phrasing and content. We selected 36 items that had a content validity index (CVI) of ≥ 0.80. The scale(S)‐CVI/Ave was 0.96.

These revised items were then subjected to a secondary round of content validation in July 2023 by a seven‐person expert panel consisting of one nursing department manager, three nursing team leaders, and three head nurses. We selected a total of 36 items, with 35 items with a CVI ≥ 0.80 and one item with a CVI of 0.71. We considered these items to be important content for measuring crisis leadership (“I have an attitude of respect towards the personal lives of nurses and patients,” in the trust factor). The S‐CVI/Ave was 0.97. We revised the phrasing and items again according to the results of the expert panel and opinions of two professors at a college of nursing. We removed three items based on opinions that they were not under the authority of nurse managers, that the content was inappropriate, or that the content related to general leadership. The remaining 33 items were selected.

The completed questionnaire was used in a pilot test with 15 nurse managers at a general hospital in July 2023. We administered an additional questionnaire to gather the participants’ opinions on the tool, including their perceptions of its effectiveness in measuring crisis leadership, any unclear words or phrases, their overall understanding of the items, and any content that may have been ambiguous. After revising an ambiguous term (Item 3: provision of resources‐ > provision of inventory), the final 33 items for the provisional tool were confirmed.

### 2.5. Validation Stage

#### 2.5.1. Participants and Data Collection

To test the validity and reliability of the provisional tool, we administered the survey in the form of a mobile or paper questionnaire to head nurses, team leaders, directors of nursing, and department managers at general and advanced hospitals in South Korea between August and October 2023. For data collection, we explained the study objectives to the nursing departments at 34 hospitals and sent public notices to each relevant organization as required. Subsequently, with the cooperation/approval of the departments, we distributed paper and mobile questionnaires. We received 452 responses. Responses were excluded if the participant selected the same response option for all items, indicating a uniform response pattern suggestive of inattentive answering. In addition, any response set with more than 10% missing data—defined as more than three unanswered items out of the total 28—was excluded to maintain the integrity of the dataset. On applying these criteria, nine responses were identified as insincere or incomplete and were excluded from the final analysis, and the remaining 443 were finally included in the analysis.

#### 2.5.2. Testing Validity and Reliability

To test the construct validity, we performed item analysis by calculating the item‐total correlation; conducted exploratory and confirmatory factor analysis; and analyzed the convergent, discriminant, and criterion‐related validity.

To test the convergent and discriminant validity of the items, we obtained the composite reliability (CR) and average variance extracted (AVE) and calculated the squares of the correlation coefficients. Further, we employed Pearson’s correlation analysis to determine the correlation of crisis leadership with empowerment, which was found to be related in a previous study. The empowerment tool used in this study was originally developed by Spreitzer [28], based on the psychological empowerment theory of Thomas and Velthouse [29]. It was later translated into Korean by Jeong [30] and subsequently used in research by Nam and Park [31]. Empowerment was measured using 10 items divided into four subdomains: three items for self‐determination, three items for significance, two items for competencies, and two items for effectiveness. Each item was rated on a five‐point scale, yielding a final score of 10–50 points. Higher scores indicated higher empowerment. Cronbach’s *α* was 0.90 at the time of development and 0.88 in our study.

To determine the discriminant validity, we analyzed the correlations of the items with organizational commitment, which had previously shown a weak correlation with general leadership, using Pearson’s correlation analysis. To measure organizational commitment, we used Kim’s [32] translation of the Organizational Commitment Questionnaire, developed by Mowday et al. [33]. Seven items across three domains were used: three items on attachment, two items on identification, and two items on continuing work. Each item was rated on a 5‐point scale, yielding a final score of 7–35 points. Higher scores indicated a greater degree of organizational commitment. Cronbach’s *α* was 0.85 at the time of development and 0.88 in our study.

For criterion validity, we analyzed the correlation of the items with the ILS, which is used to measure general crisis leadership. The ILS was developed by Aarons et al. [20] and consists of 12 items on leadership support for the use of evidence‐based practices. We used it to test criterion validity because of its predominant use in acute care settings and mental care settings overseas. The ILS is divided into four subscales: proactive leadership, knowledgeable leadership, supportive leadership, and persevering leadership. Each item is scored on a Likert‐type scale from 0 points (strongly disagree) to 4 points (strongly agree). The total ILS score is calculated as the average of the scores for the four subscales. The original English version of the ILS was translated into Korean by a Doctor of Nursing who is proficient in English, and a back‐translation was produced by a native English speaker with a master’s degree. The final translation was then produced through a process of gradual refinements by comparing the original translation with the back‐translation. Cronbach’s *α* was 0.98 at the time of original development and 0.91 in our study.

### 2.6. Ethical Considerations

Before commencing data collection, we received approval from the Institutional Review Board of Chungnam National University Se‐Jong Hospital (approval number: CNUSH 2023‐03‐006‐006). With the cooperation of leaders at each institution, we received permission from the departments of nursing to include their nurse managers in our study. Online consent forms and questionnaires were sent to the nursing departments, who distributed them to the nurse managers. The participants completed the survey either via a mobile link (distributed through SMS or email) or a paper‐based questionnaire. The completed questionnaires could only be examined by the principal investigators. We determined that the participants would have no problem in voluntarily deciding whether to participate in the study or understanding the content of the remote consent form and questionnaire; hence, we proceeded with online consent forms and questionnaires alongside paper‐based forms and questionnaires. To reduce social desirability bias, we assured participants of anonymity and confidentiality. The participants were informed that their responses would be used solely for research purposes and would not affect their evaluations or professional status.

### 2.7. Data Analysis

The data in this study were analyzed using SPSS/WIN 22.0 (IBM Corp., Armonk, NY, USA) [34] and AMOS 20.0 (IBM Corp.) [35]. We analyzed the frequency of the general characteristics of the participants. For item analysis, we calculated the coefficients of the correlation of each item with the overall score. We also examined whether the data were suitable for factor analysis based on the Kaiser–Meyer–Olkin (KMO) statistic and Bartlett’s test of sphericity. We extracted factors using exploratory principal component analysis to test construct validity and applied varimax rotation. We also extracted items with factor loading ≥ 0.4. The structural relationships of the factors derived in the exploratory factor analysis were verified using confirmatory factor analysis. Considering that the skewness and kurtosis values of the individual items met the assumption of normality, we used maximum likelihood estimation for analysis. We used absolute and incremental fit indices to test the fit of the research model and an alternative model, and we analyzed the correlations among the factors, CR, and AVE.

To determine criterion validity, we calculated Pearson’s coefficient of the correlation between the tool developed in this study and the ILS. To determine the reliability of the newly developed tool, we analyzed the internal consistency of the items using Cronbach’s α.

## 3. Results

### 3.1. General Characteristics

The sample included five male (1.1%) and 438 female participants (98.9%). The most common age group was 40–49 years (50.1%). Regarding educational attainment, 12 participants (2.7%) had graduated from professional colleges, 90 (20.3%) had an undergraduate degree, and 341 (77.0%) had a graduate degree or higher. The most common work department was the internal medicine ward (104 participants, 23.5%), the most common position was head nurse (328; 74.0%), the most frequent duration of clinical experience was 23–30 years (135; 30.5%), and the most frequent duration of managerial experience was 5–10 years (108; 24.4%). Overall, 384 participants (86.7%) were married, and 229 (51.7%) adhered to a religion. The most common hospital type was general hospital (264 individuals, 59.6%). The scale of the hospital was 500–1000 beds for 224 participants (50.6%); 288 (65%) participants reported having experienced a hospital crisis, and 222 (50.1%) experienced an infectious disease outbreak (Table 1).

**Table 1:** General characteristics of participants (*N* = 443).

Characteristics	Categories	*N*	(%)
Sex	Male	5	1.1
	Female	438	98.9

Age (years)	30–39	15	3.4
	40–49	222	50.1
	50–60	206	46.5

Educational level	College	12	2.7
	University	90	20.3
	Graduate school or above	341	77.0

Working site	Internal ward	104	23.5
	Surgical wards	83	18.7
	Intensive care unit	42	9.5
	Emergency room	24	5.4
	Operating room	17	3.8
	Department of Nursing	77	17.4
	Outpatient department	32	7.2
	Examination room	4	0.9
	Infection Control Office	8	1.8
	Nonclinical department	25	5.6
	Delivery room	7	1.6
	Dialysis department	13	2.9
	Anesthesiology department	7	1.6

Position	Head nurse	328	74.0
	Team leader	90	20.3
	Nursing supervisor level	20	4.5
	Nursing director level	5	1.1

Total clinical experience (years)	< 10	4	0.9
	10–15	10	2.3
	15–20	42	9.5
	20–25	127	28.7
	25–30	135	30.5
	> 30	125	28.2

Nursing manager experience (years)	< 3	112	25.3
	3–5	88	19.9
	5–10	108	24.4
	10–15	72	16.3
	15–20	35	7.9
	> 20	28	6.3

Marital status	Unmarried	54	12.2
	Married	384	86.7
	Divorce	5	1.1

Religion	Yes	229	51.7
	No	214	48.3

Level of hospital	General hospital	264	59.6
	Advanced general hospital	167	37.7
	Other medical institutions	12	2.7

Hospital bed size	< 200	11	2.5
	200–300	52	11.7
	300–500	102	23.0
	500–1000	224	50.6
	> 1000	54	12.2

Crisis situation experience	Yes	288	65.0
	No	155	35.0

Crisis situation experienced	Infectious disease pandemic	222	50.1
	Natural disasters	7	1.6
	Accident/disaster/fire	7	1.6
	Manpower shortage	21	4.7
	Patient safety accidents in hospitals	28	6.3
	Facilities and supplies	1	0.2
	Hospital opening/certification evaluation	1	0.2
	Department adaptation	1	0.2
	None	155	35.0

### 3.2. Construct Validity

#### 3.2.1. Exploratory Factor Analysis

We conducted a survey using the 33 provisional items selected through content validity testing with an expert panel. We tested the construct validity to verify whether the tool had formed a suitable factor structure. Before conducting exploratory factor analysis, we analyzed the correlation of each item with the total score. None of the items had a revised item‐total correlation coefficient of ≤ 0.3; hence, no item was deleted. Regarding the reliability of the 33‐item tool, Cronbach’s *α* was 0.958. The range of skewness of each item was −1.10–0.19 and that of kurtosis was −0.71–0.40; these ranges were within the thresholds of ±3 for skewness and ±8 for kurtosis, respectively, satisfying the assumption of normality. On examining whether the structure was suitable for factor analysis, the KMO statistic was 0.944, and Bartlett’s test of sphericity was statistically significant (*x* ^2^ = 4275.09, *p* < 0.001); therefore, we determined that the data were suitable.

In the principal component analysis, no item had a communality of ≤ 0.4; however, Item 20 (factor loading, 0.383) was removed owing to a factor loading of ≤ 0.4. Items CL21, CL1, CL14, and CL29 were retained despite having slightly lower factor loadings (< 0.5) owing to their conceptual relevance. These items reflect the core aspects of crisis leadership, such as staff prioritization, managing goods, providing information, and empathy. When we repeated the analysis, no item had a communality of ≤ 0.4. However, of the six derived factors, Item 13 in Factor 6 was judged to fit Factor 3 better semantically, and because Factor 6 only consisted of Item 12, Item 12 was deleted. After deleting Items 20 and 12, the analysis was repeated, resulting in 31 items and 5 factors. Considering the semantic content of the items and the authors’ judgment, we moved Item 15 from Factor 2 to Factor 3 because the factor loading difference was < 0.2 and the item was judged to be more closely related to Factor 3 than Factor 2. Items 30, 31, and 32 also showed a significant difference in factor loading of < 0.2 between Factors 1 and 3 but were judged to be closer in content to Factor 1 than to Factor 3; hence, they were included in Factor 1. Item 21 showed a high factor loading of ≥ 0.5 in Factors 3 and 5 but was judged to be an essential item for crisis leadership and to be better suited to Factor 5 than to Factor 3; hence, it was included in Factor 5 (Table 2).

**Table 2:** Exploratory factor analysis (*N* = 220).

Item	1	2	3	4	5
CL24	0.744	0.166	0.190	0.122	0.163
CL25	0.733	0.190	0.079	0.233	0.184
CL26	0.671	0.326	0.260	0.203	0.153
CL27	0.671	0.282	0.215	0.165	0.208
CL23	0.652	0.263	0.241	0.232	0.124
CL33	0.558	0.264	0.382	0.136	0.064
CL22	0.538	0.330	0.169	0.331	0.165
CL21	0.485	0.430	0.052	0.307	0.229
CL31	0.463	0.082	0.566	0.147	0.196
CL30	0.406	0.197	0.546	0.238	0.212
CL32	0.494	0.353	0.521	0.045	0.042
CL9	0.187	0.782	0.115	0.160	0.179
CL10	0.253	0.716	0.132	0.101	0.205
CL8	0.233	0.654	0.187	0.198	0.266
CL11	0.304	0.586	0.230	0.040	0.106
CL6	0.283	0.563	0.281	0.304	0.083
CL7	0.221	0.549	0.383	0.290	0.252
CL1	0.335	0.408	0.254	0.247	0.139
CL14	0.102	0.372	0.642	0.088	0.256
CL13	0.180	0.278	0.600	0.298	0.075
CL16	0.251	0.386	0.513	0.220	0.048
CL15	0.240	0.467	0.374	0.250	0.125
CL4	0.268	−0.030	0.153	0.775	0.171
CL3	0.194	0.304	0.127	0.749	0.049
CL5	0.188	0.320	0.215	0.580	0.262
CL2	0.208	0.284	0.238	0.559	0.122
CL19	0.169	0.279	−0.118	0.154	0.738
CL18	0.161	0.241	0.322	0.151	0.685
CL17	0.188	0.388	0.289	0.048	0.655
CL28	0.260	−0.012	0.511	0.217	0.552
CL29	0.358	0.056	0.410	0.266	0.491
Eigenvalue	5.002	4.681	3.637	2.994	2.752
Common variance (%)	16.135	15.100	11.732	9.658	8.876
Cumulative variance (%)	16.135	31.235	42.967	52.625	61.501

*Note:* KMO = 0.945, Bartlett’s *x* ^2^ = 4024.61 (*p* < 0.001).

The KMO statistic of the final model was high, at 0.945, and Bartlett’s test of sphericity was statistically significant (*x* ^2^ = 4024.61 *p* < 0.001). There were 31 items in the five factors with eigenvalues of ≥ 1, and the communality was ≥ 0.423. The model explained 61.50% of the total input variables, and the factor loading was ≥ 0.408 (Table 2). Based on the content and properties of the included items, the factors of crisis leadership were named as follows: Factor 1 was “crisis management competencies,” Factor 2 was “decision making,” Factor 3 was “collaboration,” Factor 4 was “communication,” and Factor 5 was “building trust.”

#### 3.2.2. Confirmatory Factor Analysis

**Figure 2:**
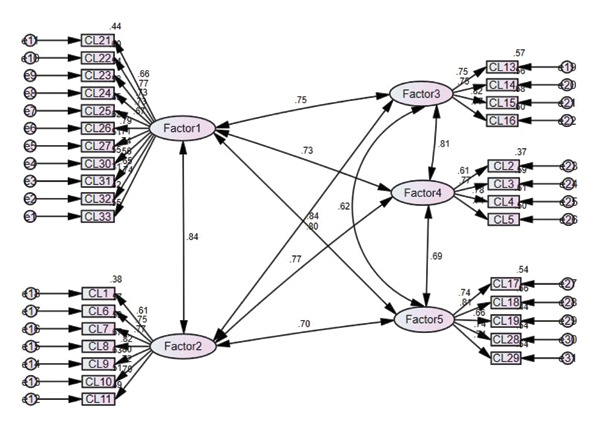
Proposed model of crisis leadership.

We performed confirmatory factor analysis to examine the structural relationships of the five factors derived in exploratory factor analysis. The *p* values of the regression weights were all significant, and all of the standardized regression weights were ≥ 0.5 (Figure 2). For Items 31, 2, and 1, which had squared multiple correlation (SMC) values < 0.4, we tested the model fit while removing the items in order of the smallest SMC first. In terms of model fit, the *x* ^2^/df was 2.092, which was < 3, Tucker–Lewis index (TLI) was 0.893, and comparative fit index (CFI) was 0.903. Meanwhile, the root mean square error of approximation (RMSEA) was 0.081, and the standardized root‐mean‐square residual (SRMR), at 0.07, was < 0.08, suggesting that some fit indices did not show a good fit. The correlation between factors was high, at 0.69–0.84 (Figure 2), and the correlation was especially high between Factors 1 and 2 and between Factors 1 and 5. Therefore, considering the semantic content of the items, we moved Items 29 and 28 to Factor 1 and repeated the analysis.

**Figure 3:**
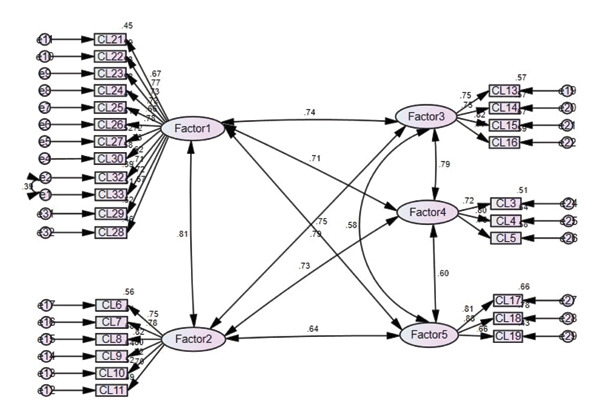
Modified model of crisis leadership.

Regarding the revised indices, the covariance between e1 and e2 was high, at 31.692; thus, after setting the correlations, the final fit indices were CMIN/DF = 1.965, TLI = 0.905, CFI = 0.915, RMSEA = 0.066, and SRMR = 0.023; the 90% confidence interval of the RMSEA was 0.059–0.073, which, being ≤ 0.10, indicated good fit for our model. The correlations among the factors were in the range of 0.579–0.815 (Figure 3).

#### 3.2.3. Convergent and Discriminant Validity

The results of convergent and discriminant validity analyses are as follows. The standardized path coefficients (β) for each factor were high, at 0.624–0.82 (Table S1). To ensure convergent validity, the CR should be ≥ 0.70 and the AVE should be ≥ 0.05. For two factors, these criteria were satisfied, demonstrating the convergent validity of the items (Table S2).

To demonstrate discriminant validity, the AVE of each factor should be larger than the square of the correlation coefficient with other factors. The smallest AVE value of 0.69 was larger than the largest *r* ^2^ value of 0.82, demonstrating the discriminant validity of the items (Table S2). Another criterion for discriminant validity is that the correlation coefficient should be greater than two standard errors from 1, and this was the case for all pairs of factors, further demonstrating the discriminant validity (Table 3).

**Table 3:** Discriminant validity.

	Factor 1	Factor 2	Factor 3	Factor 4	Factor 5
Factor 1	1				
Factor 2 (*ρ* + 2 ∗ S.E.–*ρ* − 2 ∗ S.E.)	0.761–0.869	1			
Factor 3 (*ρ* + 2 ∗ S.E. − *ρ* − 2 ∗ S.E.)	0.683–0.791	0.733–0.841	1		
Factor 4 (*ρ* + 2 ∗ S.E.–*ρ* − 2 ∗ S.E.)	0.668–0.76	0.683–0.771	0.739–0.835	1	
Factor 5 (*ρ* + 2 ∗ S.E.–*ρ* − 2 ∗ S.E.)	0.703–0.803	0.593–0.681	0.533–0.625	0.565–0.641	1

To test the convergent validity, we analyzed the correlation between crisis leadership and empowerment, which have been shown to be related in previous studies. We observed a significant moderate correlation of empowerment with each of the factors in crisis leadership (*r* = 0.426–0.614, *p* < 0.01; Table S3).

We also analyzed the relationship between crisis leadership and organizational commitment, which had shown a weak correlation in previous studies, as a test of discriminant validity, and we observed a significant and weak positive correlation (*r* = 0.284–0.301, *p* < 0.01; Table S4).

#### 3.2.4. Criterion Validity

In our study, the correlation between our crisis leadership measurement tool and the ILS general leadership tool was in the range of 0.279–0.406, showing a significant positive correlation of weak‐to‐moderate strength. Further, the correlations with the ILS were 0.390 for Factor 1, 0.343 for Factor 2, 0.340 for Factor 3, 0.279 for Factor 4, and 0.285 for Factor 5, and the correlation with overall crisis leadership was 0.406. All correlations were statistically and significantly weak‐to‐moderate (Table 4).

**Table 4:** Correlation between relevant constructs of crisis leadership and ILS.

	Factor 1	Factor 2	Factor 3	Factor 4	Factor 5	Total crisis leadership	ILS
Factor 1	1						
Factor 2	0.694^∗∗^	1					
Factor 3	0.656^∗∗^	0.645^∗∗^	1				
Factor 4	0.664^∗∗^	0.582^∗∗^	0.678^∗∗^	1			
Factor 5	0.634^∗∗^	0.548^∗∗^	0.544^∗∗^	0.498^∗∗^	1		
Total crisis leadership	0.940^∗∗^	0.841^∗∗^	0.809^∗∗^	0.780^∗∗^	0.731^∗∗^	1	
ILS	0.390^∗∗^	0.343^∗∗^	0.340^∗∗^	0.279^∗∗^	0.285^∗∗^	0.406^∗∗^	1

Abbreviation: ILS, Implementation Leadership Scale.

^∗∗^ *p* < 0.01.

### 3.3. Reliability Testing and Confirmation of the Final Measurement Tool

The Cronbach’s α of the measurement tool developed in this study was 0.96, indicating the overall reliability of the measurement tool, and the reliability of each factor was relatively high at 0.92 for Factor 1, 0.88 for Factor 2, 0.83 for Factor 3, 0.79 for Factor 4, and 0.80 for Factor 5. The crisis leadership measurement tool was finalized with five factors and 28 items. Each item was measured on a 5‐point Likert scale from 1 point (“Never”) to 5 points (“Always”). The range of possible scores was 28–140 points, and higher total scores indicated a higher level of crisis leadership demonstrated by nurse managers.

## 4. Discussion

In the tool development stage, leadership was divided into six factors of “communication,” “decision‐making,” “collaboration,” “information sharing,” “trust,” and “competencies,” and these were confirmed empirically in the evaluation stage. In factor analysis to test the construct validity, the factors were classified as follows: Factor 1 was “crisis management competencies,” Factor 2 was “decision making,” Factor 3 was “collaboration,” Factor 4 was “communication,” and Factor 5 was “building trust.” The factor of “information sharing” generated in the development stage was combined with the factor of communication.

In a previous study that developed a tool to measure the self‐efficacy of leaders in a crisis, learning goal orientation, intelligence, and divergent thinking ability were reported to increase a leader’s self‐efficacy, enabling them to appropriately respond to the crisis [19]. These properties are similar to the factor of crisis management competencies in our tool, indicating that improved crisis management competencies can lead to improved crisis leadership.

Another study on leadership during crises emphasized the importance of forming connections by listening closely to the concerns of staff, empathizing, and providing support [36]. These elements were included as items in the communication and crisis management factors in our tool. Thus, the items in our tool included suitable crisis leadership properties.

When we tested the convergent validity of our tool, we observed significant moderate positive correlations between empowerment and each factor of crisis leadership (*r* = 0.426–0.614, *p* < 0.01), and many properties of empowerment (self‐confidence in work performance abilities, job skills, and self‐determination of job performance) were included in the crisis leadership tool (self‐confidence, skills‐crisis management competencies, and self‐determination‐decision making).

To test the discriminant validity, we examined organizational commitment (attachment, identification, and continuing work), which had shown a weak correlation with general leadership in previous studies [37], and we observed a significant weak positive correlation with crisis leadership (*r* = 0.284–0.301, *p* < 0.01). This aspect demonstrates that the items included in the factors in our tool for measuring crisis leadership were differentiated from the items included for measuring organizational commitment. Additionally, as a measure of the discriminant validity of the items, the AVE of each factor was greater than the squares of the correlation coefficients between the factors, implying that it was appropriate to calculate individual scores for each factor.

The ILS, a tool for measuring general leadership, consists of four subscales: Proactive leadership, knowledgeable leadership, supportive leadership, and perseverant leadership. Since the factors in proactive leadership included planning based on general evidence‐based practice, eliminating obstacles/difficulties, and establishing departmental standards, our tool also included factors for increasing time efficiency and work efficiency specifically in a crisis scenario. Similarly, aspects of knowledgeable leadership such as possessing knowledge about evidence‐based practice, being able to educate staff, and implementing these practices were also included in our tool. However, our tool additionally included factors, such as clear communication and decision‐making in a crisis. Supportive leadership includes factors such as supporting the efforts of staff to learn and use evidence‐based practice and noticing and showing gratitude for successful work performance, which are also included in our tool (building trust). However, our tool included supporting patients and staff in a crisis. Aspects of perseverant leadership, such as the ups and downs and challenges of evidence‐based practice and facing clinical issues for effective problem‐solving, were included. Our tool also included the aspect of cooperation with the department and hospital to solve problems in a crisis. The correlation coefficients of crisis leadership with the ILS were 0.279–0.406, showing a significant weak‐to‐moderate positive correlation. Therefore, as a tool that was differentiated from the ILS, the criterion validity of our tool was established.

This study has a few limitations. As this study involved nurse managers from 34 hospitals within a specific region; hence, the findings may not be generalizable to nurse managers in different settings or countries. To minimize the social desirability bias, the survey was conducted anonymously, and participants were assured that their responses would remain confidential and would not affect their professional evaluations. Nonetheless, as with most self‐reported measures, the possibility of overestimation cannot be fully excluded. Future studies should consider integrating objective performance assessments or peer evaluations to complement self‐perception scores.

## 5. Conclusions

We developed a 28‐item crisis management tool (Table S5) to enable quantitative analysis of crisis leadership among nurse managers and demonstrated its validity and reliability. The tool exhibited a Cronbach’s *α* of 0.96, indicating strong internal consistency. Our tool can provide nurse managers and hospital administrators with a structured approach to evaluate and enhance leadership during crises. It can be applied in various clinical settings to identify leadership gaps, guide training programs, and improve crisis management protocols, ultimately enhancing patient care and safety. We propose the following recommendations for further studies: First, to verify the validity of our tool and assess its stability, cross‐validation studies with nurse managers from diverse regions or healthcare organizations are needed. Second, we recommend active use of our tool in clinical settings to develop and validate nursing intervention programs aimed at improving the crisis leadership performance of nurse managers. Finally, longitudinal studies using our tool are needed to further analyze differences in coping strategies, resilience, and recovery from crises based on the level of crisis leadership performance.

## Disclosure

A portion of this work was previously presented as a poster at the 2024 Gerontological Society of America (GSA) Annual Scientific Meeting [38].

## Conflicts of Interest

The author declares no conflicts of interest.

## Funding

This research received no specific grant from any funding agency in the public, commercial, or not‐for‐profit sectors.

## Supporting Information

Table S1. Composite reliability and average variance extracted of confirmatory factor analysis (*N* = 223)

Table S2. Correlation and reliability analysis of constructs.

Table S3. Correlation between relevant constructs of crisis leadership and empowerment: convergent validity.

Table S4. Correlation between relevant constructs of crisis leadership and organizational commitment: discriminant validity.

Table S5. Crisis Leadership Measurement Instrument.

## Supporting information


**Supporting Information** Additional supporting information can be found online in the Supporting Information section.

## Data Availability

The data that support the findings of this study are available on request from the corresponding author. The data are not publicly available due to privacy or ethical restrictions.
