# Supplementary material for: Development and Validation of a Self‐Perception Tool for Measuring Nurse Managers’ Crisis Leadership
Source: J Nurs Manag. 2025 Dec 6;2025:3754050. doi: 10.1155/jonm/3754050 (PMC12714172; doi:10.1155/jonm/3754050)
Supplement: Supplementary file 1 — Supporting Information Additional supporting information can be found online in the Supporting Information section. [file JONM-2025-3754050-s001.docx]

**SUPPLEMENTARY TABLES**

**Table S1**. CR and AVE of Confirmatory Factor Analysis (N=223)

|  | **Items** | **Estimate** | | **SE** | **CR** |
| --- | --- | --- | --- | --- | --- |
|  |  | **B** | **β** |  |  |
| **Factor 1** | CL33 | 1 | 0.715 |  |  |
|  | CL32 | 0.841 | 0.624 | 0.073 | 11.476 *** |
|  | CL30 | 0.961 | 0.76 | 0.087 | 11.041 *** |
|  | CL27 | 0.973 | 0.721 | 0.093 | 10.463 *** |
|  | CL26 | 1.097 | 0.783 | 0.096 | 11.375 *** |
|  | CL25 | 1.079 | 0.664 | 0.112 | 9.627 *** |
|  | CL24 | 1.104 | 0.723 | 0.105 | 10.497 *** |
|  | CL23 | 1.014 | 0.726 | 0.096 | 10.541 *** |
|  | CL22 | 1.039 | 0.77 | 0.093 | 11.175 *** |
|  | CL21 | 0.828 | 0.669 | 0.085 | 9.707 *** |
|  | CL29 | 1.022 | 0.724 | 0.097 | 10.509 *** |
|  | CL28 | 0.819 | 0.675 | 0.084 | 9.791 *** |
| **Factor 2** | CL11 | 1 | 0.701 |  |  |
|  | CL10 | 1.052 | 0.724 | 0.104 | 10.148 *** |
|  | CL9 | 1.063 | 0.799 | 0.095 | 11.152 *** |
|  | CL8 | 1.056 | 0.823 | 0.092 | 11.467 *** |
|  | CL7 | 0.948 | 0.779 | 0.087 | 10.894 *** |
|  | CL6 | 1.06 | 0.748 | 0.101 | 10.47 *** |
| **Factor 3** | CL13 | 1 | 0.754 |  |  |
|  | CL14 | 0.872 | 0.752 | 0.078 | 11.118 *** |
|  | CL15 | 1.001 | 0.82 | 0.082 | 12.177 *** |
|  | CL16 | 0.889 | 0.771 | 0.078 | 11.409 *** |
| **Factor 4** | CL3 | 1 | 0.783 |  |  |
|  | CL4 | 1.098 | 0.804 | 0.094 | 11.698 *** |
|  | CL5 | 0.766 | 0.709 | 0.074 | 10.333 *** |
| **Factor 5** | CL17 | 1 | 0.813 |  |  |
|  | CL18 | 1.148 | 0.884 | 0.084 | 13.652 *** |
|  | CL19 | 0.918 | 0.655 | 0.092 | 10.027 *** |

SE=standard error; CR=critical ratio; *** *p* < .001

**Table S2**. Correlation and Reliability Analysis of Constructs

|  | **Factor 1** | **Factor 2** | **Factor 3** | **Factor 4** | **Factor 5** | **AVE** | **CR** |
| --- | --- | --- | --- | --- | --- | --- | --- |
| **Factor 1 (**$\boldsymbol{\rho}^{\boldsymbol{2}}$**)** | 1 |  |  |  |  | 0.69 | 0.96 |
| **Factor 2 (**$\boldsymbol{\rho}^{\boldsymbol{2}}$**)** | 0.82 (.66) ** | 1 |  |  |  | 0.78 | 0.95 |
| **Factor 3 (**$\boldsymbol{\rho}^{\boldsymbol{2}}$**)** | 0.737 (.54) ** | 0.787 (.62) ** | 1 |  |  | 0.8 | 0.94 |
| **Factor 4 (**$\boldsymbol{\rho}^{\boldsymbol{2}}$**)** | 0.714 (.51) ** | 0.727 (.53) ** | 0.787 (.62) ** | 1 |  | 0.78 | 0.91 |
| **Factor 5 (**$\boldsymbol{\rho}^{\boldsymbol{2}}$**)** | 0.753 (.57) ** | 0.637 (.41) ** | 0.579 (.34) ** | 0.603 (.36) ** | 1 | 0.82 | 0.93 |

AVE=average variance extract; CR=construct reliability; ρ=correlation coefficient

***p* < .01

**Table S3**. Correlation between relevant constructs of crisis leadership and empowerment: convergent validity

|  | **Factor 1** | **Factor 2** | **Factor 3** | **Factor 4** | **Factor 5** | **TOTAL Crisis Leadership** | **Empowerment** |
| --- | --- | --- | --- | --- | --- | --- | --- |
| **Factor 1** | 1 |  |  |  |  |  |  |
| **Factor 2** | .707^**^ | 1 |  |  |  |  |  |
| **Factor 3** | .666^**^ | .656^**^ | 1 |  |  |  |  |
| **Factor 4** | .672^**^ | .588^**^ | .686^**^ | 1 |  |  |  |
| **Factor 5** | .640^**^ | .556^**^ | .543^**^ | .506^**^ | 1 |  |  |
| **TOTAL Crisis Leadership** | .942^**^ | .848^**^ | .813^**^ | .784^**^ | .733^**^ | 1 |  |
| **Empowerment** | .603^**^ | .511^**^ | .438^**^ | .426^**^ | .516^**^ | .614^**^ | 1 |

***p* < .01

**Table S4**. Correlation between relevant constructs of crisis leadership and organizational commitment: discriminant validity

|  | **Factor 1** | **Factor 2** | **Factor 3** | **Factor 4** | **Factor 5** | **TOTAL Crisis Leadership** | **Organizational commitment** |
| --- | --- | --- | --- | --- | --- | --- | --- |
| **Factor 1** | 1 |  |  |  |  |  |  |
| **Factor 2** | .707^**^ | 1 |  |  |  |  |  |
| **Factor 3** | .666^**^ | .656^**^ | 1 |  |  |  |  |
| **Factor 4** | .672^**^ | .588^**^ | .686^**^ | 1 |  |  |  |
| **Factor 5** | .640^**^ | .556^**^ | .543^**^ | .506^**^ | 1 |  |  |
| **TOTAL Crisis Leadership** | .942^**^ | .848^**^ | .813^**^ | .784^**^ | .733^**^ | 1 |  |
| **Organizational commitment** | .307^**^ | .227^**^ | .220^**^ | .160^*^ | .222^**^ | .292^**^ | 1 |

***p* < .01

**Table S5.** Crisis Leadership Measurement Instrument

The following statements relate to your crisis leadership. After reading each statement, please put a checkmark (✓) in the box that best matches how you act in a crisis.

| **Item** | **In a crisis, I …** | **Never** | **Rarely** | **Sometimes** | **Often** | **Always** |
| --- | --- | --- | --- | --- | --- | --- |
| **1** | Manage and provide required items at the appropriate time |  |  |  |  |  |
| **2** | Manage human resources in a timely manner |  |  |  |  |  |
| **3** | Establish response plans for crisis management |  |  |  |  |  |
| **4** | Analyze failure cases or errors and apply to the crisis response |  |  |  |  |  |
| **5** | Provide nurses and patients with education for crisis management |  |  |  |  |  |
| **6** | Apply guidelines appropriately according to the situation and environment |  |  |  |  |  |
| **7** | Apply the appropriate interpersonal skills according to the situation and environment |  |  |  |  |  |
| **8** | Care for nurses and patients with a sense of duty, responsibility, and calling |  |  |  |  |  |
| **9** | Lead nurses and patients to overcome the crisis with empathic, encouraging leadership |  |  |  |  |  |
| **10** | Judge the importance of information and use it appropriately |  |  |  |  |  |
| **11** | Adapt and apply departmental work guidelines according to constantly changing hospital policy |  |  |  |  |  |
| **12** | Participate in or support processes for information sharing |  |  |  |  |  |
| **13** | Make appropriate decisions, according to guidelines, in response to problems within the hospital and affecting public healthcare systems related to the crisis |  |  |  |  |  |
| **14** | Set priorities for work order and methods according to preset policies/guidelines and fair criteria |  |  |  |  |  |
| **15** | Plan and manage schedules according to preset policies/guidelines |  |  |  |  |  |
| **16** | Divide work according to preset policies/guidelines |  |  |  |  |  |
| **17** | Make decisions about the provision of material resources according to preset policies/guidelines |  |  |  |  |  |
| **18** | Make logical decisions about human resource problems according to preset policies/guidelines |  |  |  |  |  |
| **19** | Work collaboratively with other departments and healthcare workers |  |  |  |  |  |
| **20** | Cooperate with directives from higher-level organizations, such as the hospital control tower |  |  |  |  |  |
| **21** | Rapidly share information with other departments in the hospital via information delivery systems of each department |  |  |  |  |  |
| **22** | Clearly convey information about the public healthcare systems, hospital guidelines, and crisis situations to nurses and patients |  |  |  |  |  |
| **23** | Promptly convey my opinions to senior managers about problems relating to nurse staffing and inventory provision |  |  |  |  |  |
| **24** | Actively convey the needs and discomforts of patients; caregivers; and staff, including nurses, to senior managers |  |  |  |  |  |
| **25** | Convey improvements for needs and discomforts, as discussed with senior managers, to patients; caregivers; and staff, including nurses |  |  |  |  |  |
| **26** | Treat nurses and patients with a fair attitude |  |  |  |  |  |
| **27** | Maintain a consistent attitude towards nurses and patients |  |  |  |  |  |
| **28** | Have an attitude of respect towards the personal lives of nurses and patients |  |  |  |  |  |

1–12: Crisis management competencies, 13–18: Decision-making, 19–22: Collaboration, 23–25: Communication, 26–28: Trust
